# Arterial spin labeling reveals disordered cerebral perfusion and cerebral blood flow-based functional connectivity in primary open-angle glaucoma

**DOI:** 10.1007/s11682-023-00813-2

**Published:** 2023-11-25

**Authors:** Qian Wang, Xiaoxia Qu, Huaizhou Wang, Weiwei Chen, Yunxiao Sun, Ting Li, Jianhong Chen, Yang Wang, Ningli Wang, Junfang Xian

**Affiliations:** 1 Department of Radiology, Beijing Tongren Hospital, Capital Medical University, NO.1 of Dongjiaominxiang Street, Dongcheng District, Beijing, 100730 China; 2 Beijing Tongren Eye Center, Beijing Ophthalmology and Visual Sciences Key Laboratory, Beijing Tongren Hospital, Capital Medical University, NO.1 Dongjiaominxiang Street, Dongcheng District, Beijing, 100730 China; 3 Beijing Institute of Ophthalmology, Capital Medical University, Beijing Tongren Hospital, 17 Hougou Lane, Chongwenmen, Beijing, 100005 China; 4 Department of Gastroenterology, Beijing Tongren Hospital, Capital Medical University, Beijing, China; 5 Department of Radiology, Medical College of Wisconsin, Milwaukee, WI USA

**Keywords:** Arterial spin labeling, Cerebral blood flow, Connectivity, Magnetic resonance imaging, Primary open-angle glaucoma

## Abstract

**Purpose:**

Primary open-angle glaucoma (POAG) is a widespread neurodegenerative condition affecting brain regions involved in visual processing, somatosensory processing, motor control, emotional regulation and cognitive functions. Cerebral hemodynamic dysfunction contributes to the pathogenesis of glaucomatous neurodegeneration. We aimed to investigate cerebral blood flow (CBF) redistributed patterns in visual and higher-order cognitive cortices and its relationship with clinical parameters in POAG, and we hypothesized that CBF changes together across regions within the same functional network.

**Methods:**

Forty-five POAG patients and 23 normal controls underwent three-dimensional pseudocontinuous arterial spin labeling MRI to measure the resting-state CBF. Group comparisons of CBF and correlations between CBF changes and ophthalmological and neuropsychological indices were assessed. We determined CBF-based functional connectivity (CBFC) by calculating the correlations between specific regions and all other brain voxels and compared CBFC differences between groups.

**Results:**

The patients exhibited decreased CBF in visual cortices, postcentral gyrus, inferior parietal lobule and cerebellum and increased CBF in medial, middle, and superior frontal gyri, as well as the insula. The reduced CBF in the visual cortices positively correlated with visual field defect (*r* = 0.498, *p* = 0.001) in POAG patients, while the increased CBF in the right medial frontal gyrus was negatively associated with the visual field defect (*r* = −0.438, *p* = 0.004) and positively associated with the cup-to-disc ratio (*r* = 0.469, *p* = 0.002). POAG patients showed negative connections weakening or converting to mild positive connections, as well as positive connections converting to negative connections.

**Conclusions:**

Regional and interregional CBF properties confirmed that the aberrant brain regions extend beyond the visual pathway, including the somatosensory, emotional and cognitive networks, which highlights the importance of cerebral hemodynamic dysfunction in the pathophysiology of spreading neurodegeneration in POAG.

**Supplementary Information:**

The online version contains supplementary material available at 10.1007/s11682-023-00813-2.

## Introduction

Glaucoma is a progressive neurodegenerative disease characterized by the loss of retinal ganglion cells and their axons in the optic nerve and damage to the visual field. It is a leading cause of irreversible blindness globally and will affect 111.8 million people in 2040 (Tham et al., 2014). The prevalence of primary open-angle glaucoma (POAG) is six times higher than that of primary angle-closure glaucoma (Tham et al., 2014). This condition will become more prevalent in aging populations as a byproduct of increased life expectancy, and it is predicted to increase healthcare and social burdens in the near future. The mechanisms underlying glaucoma remain unclear; elevated intraocular pressure (IOP) is the leading risk factor for glaucoma but is not the direct cause. Mounting evidence has shown that glaucoma is a multifactorial neurodegenerative disorder that affects a wide range of ages and leads to irreversible vision loss (Kasi et al., 2019; Sen et al., 2020).

On the basis of accumulating evidence, there is now a consensus that glaucoma and Alzheimer’s disease (AD) as two outcomes of a single disease spectrum (Sen et al., 2020). A longitudinal study conducted by Lourenco CF et al. revealed that cerebrovascular dysfunction preceded obvious cognitive decline, which supports the concept that cerebrovascular dysfunction is a basic process underlying neurodegenerative disease (Brown & Thore, 2011; Lourenco et al., 2017). Vascular dysfunction may play a particularly important role in POAG. In each stage of glaucoma, the microvessels of both the retina and the optic nerve head (ONH) undergo functional and morphological changes that are independent of IOP and associated with optic neuropathy (Wareham & Calkins, 2020). In addition, the neurovascular coupling response is dysfunctional in the ONH and retina in the patients with POAG (Wareham & Calkins, 2020). Apart from the ocular vascular abnormalities, increasing evidence suggests cerebral hemodynamic dysfunction contributed to the pathogenesis of glaucomatous neurodegenerative progression. This is to be expected, since the retina and brain have the strong anatomical and physiological homologies and share similar microcirculatory characteristics (Pellegrini et al., 2020). Rather than being a separate process affecting only the eye and its immediate vessels, glaucomatous optic nerve damage may be an ocular manifestation of a more widespread vascular abnormality implicating the brain.

The ophthalmic artery, middle cerebral artery and posterior cerebral artery, which account for most of the blood supply to the orbit and visual cortex, show reduced blood flow velocities in patients with POAG as measured by transcranial Doppler (Arslan et al., 2021; Harris et al., 2013). Studies conducted by three-dimensional pseudo-continuous arterial spin labeling (3D-pCASL) have demonstrated reduced resting cerebral blood flow (CBF) and reduced visual evoked CBF in the visual cortex in patients with POAG (Wang et al., 2018; Zhang et al., 2015). A previous study by our team found, in agreement with previous studies, that reduced CBF in the visual cortex was associated with glaucomatous damage to the ONH and retina (Harris et al., 2013; Wang et al., 2018). In addition, another finding from our research established that POAG patients showed disrupted coupling between resting-state CBF and functional connectivity strength (FCS) as well as altered CBF/FCS ratios (Wang et al., 2021a). The brain regions with abnormal CBF/FCS ratios extended beyond the visual system to the salience network, default-mode network (DMN), and dorsal attention network (DAN) (Wang et al., 2021a). These findings suggest that cerebral neurovascular coupling dysfunction exists in patients with POAG, and the decreased CBF/FCS ratio was caused more by cerebral hypoperfusion than by altered FC, suggesting that cerebrovascular dysfunction plays a dominant role in the dysfunction of neurovascular units (Wang et al., 2021a). We also detected malfunctions of neurovascular coupling in early POAG (Wang et al., 2021a). These findings suggest that CBF values could serve as neurological biomarker for glaucoma.

Noninvasive CBF measurement by 3D-pCASL provides capillary-level resolution, effectively represents cerebral metabolism associated with neural activity, and shows good test–retest repeatability. It enables the investigation of pathogenetic mechanisms, early detection, and monitoring of disease progression (Alsop et al., 2015; Nakamura et al., 2022). Although there have been reports on the changes in CBF of brain regions extending beyond the visual system in POAG, they have been far from sufficient. Behavior and cognition produced by cortical area interactions, which needs integration between cortical areas determining by the brain connections (Thiebaut de Schotten & Forkel, 2022). Brain pathologies amplify the connectivity differences among humans through disconnections and, consequently, the disintegration of cognitive functions (Thiebaut de Schotten & Forkel, 2022). Therefore, different from the previous studies, the current study aims to achieve three objectives: (1) to provide clarity on the patterns of CBF alterations in POAG, (2) to examine the relationships between CBF alterations and clinical parameters in POAG, and (3) to assess whether brain regions exhibiting altered CBF also demonstrate changes in CBF-based functional connectivity (CBFC) in POAG.

## Materials and methods

### Participants

Forty-five patients with POAG and 23 normal controls (NCs) were enrolled in this study; their demographics are shown in Table 1. All participants were right-handed. Patients with POAG were recruited from the inpatient and outpatient clinics of our hospital.

**Table 1:** Demographic, ophthalmological and neuropsychological data of the POAG and NC groups

Clinical-demographic characteristics	POAG patients	NCs	*P*
Age, mean ± SD (years)	43.1 ± 13.0	43.2 ± 10.5	0.973
Gender, male/female	22/23	11/12	0.934
MD, mean ± SD(dB)	−9.0 ± 6.4	−1.7 ± 1.6	<0.001
PSD, mean ± SD(dB)	6.4 ± 3.0	2.2 ± 1.3	<0.001
CDR, mean ± SD(μm)	0.7 ± 0.2	0.3 ± 0.1	<0.001
BDI	10.4 ± 8.8	3.4 ± 3.2	<0.001
STAI-T	41.4 ± 9.7	31.8 ± 8.4	<0.001
STAI-S	39.4 ± 11.0	30.4 ± 8.5	0.001
MoCA	25.6 ± 3.3	26.0 ± 2.1	0.467
SDMT	53.3 ± 13.2	53.0 ± 10.6	0.917

Abbreviations: *POAG* primary open angle glaucoma, *NCs* normal controls, *SD* standard deviation, *MD* mean deviation of visual field defect, *PSD* pattern standard deviation of visual field defect, *CDR* cup-to-disc ratio, *BDI* beck depression inventory, *STAI-S and STAI-T*, state-trait anxiety inventory, *MoCA* montreal cognitive assessment, *SDMT* symbol digit modalities test

The POAG patients were enrolled according to the following inclusion criteria: (1) age 30 to 65 years and (2) a clinical examination confirming POAG. Three glaucoma specialists evaluated the examination results while blinded to patient information, and participants were enrolled in the study only if all three specialists agreed on the diagnosis. The exclusion criteria for POAG patients as well as NCs were (1) clinical evidence or history of other oculopathy; (2) presence of significant psychiatric, neurological, or systemic comorbidity; (3) previous or active cerebrovascular or neurodegenerative diseases, intracranial tumors, or previous cranial surgery; and (4) contraindications to MRI scanning.

### Ophthalmological examinations and neuropsychological assessments

The detailed ophthalmologic examination included visual acuity, refraction, slit-lamp biomicroscopy, applanation tonometry, gonioscopy, dilated fundus examinations, OCT, and visual field (VF) measurement. The visual field defect was performed using the Humphrey Visual Field Analyzer with a central 30–2 full-threshold program (Zeiss Meditec AG, Jena, Germany). Standard Automated Perimetry examinations were considered reliable in the presence of fixation errors < 20%, false-positive results < 15%, and false-negative results < 33%. Spectral domain OCT (RTVue-100, software version 4.0; Optovue Inc., Fremont, CA) was used to measure the cup-to-disc ratio (CDR).

Neuropsychological tests were performed for all participants. The Montreal Cognitive Assessment, MoCA) was used to roughly assess cognitive function (Nasreddine et al., 2005). The Symbol Digit Modalities Test (SDMT) was used to measure information processing speed and working memory (Pascoe et al., 2018). The Beck Depression Inventory (BDI) (Beck et al., 1961) and State-Trait Anxiety Inventory (STAI-T/S) (Loo,  1979)were used to evaluate depression and anxiety, respectively.

### MRI data acquisition

Images were collected with a 3.0-T MR scanner (Discovery MR750; GE Healthcare, Milwaukee, WI, USA) using an 8-channel head coil. The MRI protocol included structural 3D T1-weighted imaging (T1WI) with the following acquisition parameters: repetition time (TR) = 8.16 ms, echo time (TE) = 3.18 ms, inversion time (TI) = 450 ms, flip angle = 12°, matrix = 256 × 256, thickness = 1.0 mm, gap = 0 mm, slices = 188 and voxel size = 1 × 1 × 1 mm3. The perfusion images were obtained using a pCASL sequence with a 3D fast spin‒echo acquisition and background suppression with the following parameters: TR = 5046 ms; TE = 10.5 ms; labeling time = 1450ms; post-labeling delay (PLD) = 2025 ms; spiral in readout of 8 arms with 512 sample points; field of view = 240 × 240 mm^2^; reconstruction matrix = 128 × 128; slice thickness = 3 mm, no gap; axial slices = 50; number of excitations = 3. The CBF automatic generation option was selected during ASL scanning, and CBF images were acquired from the raw ASL data obtained by the MRI scanner. Finally, each subject contained 50 control-labeled image pairs and subsequently acquired 50 CBF images calculated automatically by the MRI scanner using the following formula (Alsop et al., 2015):

$$CBF=\frac{6000\cdot\lambda\cdot\left({SI}_{control}-{SI}_{label}\right)\cdot e^\frac{PLD}{T_{1,blood}}}{2\cdot\alpha\cdot T_{1,blood}\cdot{SI}_{PD}\cdot\left(1-e^{-\frac\tau{T_{1,blood}}}\right)}\left[ml/100g/min\right]$$

where the factor of 6000 is a constant value, used to converts the units from mL/g/s to mL/ (100 g)/min. λ is the brain/blood partition coefficient in mL/g, ${SI}_{control}$ and ${SI}_{label}$ are the time-averaged signal intensities in the control and label images, respectively. ${T}_{1, blood}$ signifies the longitudinal relaxation time of blood in seconds, $\alpha$ is the labeling efficiency, ${SI}_{PD}$ is the signal intensity of a proton density-weighted image, and $\tau$ is the label duration. $PLD$ is the post-labeling delay time.

During the scans, subjects were instructed to lie quietly with their eyes closed, try to relax, remain as still as possible, think about nothing, and avoid falling asleep. Foam padding and earplugs were used to limit head movement and attenuate scanner noise input, respectively.

### CBF analysis and correlations between CBF and clinical indices

CBF images were used for data processing. SPM12 software (SPM, http://www.fil.ion.ucl.ac.uk/spm) running in MATLAB (MATLAB R2016a; MathWorks, Inc., Natick, MA) was used to preprocess the CBF images by the following steps: (1) The CBF images of all participants were coregistered to their structural images (3D T1WI). (2) Individual structural 3D T1WI images were normalized to Montreal Neurological Institute (MNI) space using the deformation fields generated during segmentation and normalization. (3) All the CBF images were converted to MNI space using the deformation parameter derived from step (2) and were resliced into a 3 mm × 3 mm × 3 mm voxel size. (4) The CBF maps were further standardized. (5) Standardized CBF maps were spatially smoothed with a Gaussian kernel with a 6 mm full width at half maximum.

Group differences in CBF were compared between POAG patients and NCs by using voxelwise independent-sample t-tests with age, sex, and years of education as the nuisance variables. Multiple comparisons were corrected using a voxelwise Gaussian random field (GRF) theory correction method with a voxel-level* p* < 0.001 and a cluster-level *p* < 0.05. For each participant, the mean CBF value of each cluster with significant group differences was extracted for the subsequent region of interest (ROI)-based analyses (correlation study and CBFC calculation).

Pearson correlation analyses were used to test the associations between the CBF value of each significantly changed cluster and the mean deviation (MD), CDR and neuropsychological scores of the POAG patients. The Bonferroni method was applied to correct for multiple comparisons (*p* < 0.05/11 = 0.005).

### CBF connectivity analysis

The computation of the correlation coefficient between the CBF values of two distinct brain regions within a cohort of subjects serves to elucidate the co-variation properties of CBF across different brain regions, which can be understood as the CBF connectivity between brain regions (Melie-Garcia et al., 2013; Zhu et al., 2015). To test whether the brain regions with aberrant CBF had altered CBFC in POAG, the differences in CBFC patterns within the same ROIs between POAG patients and NCs were investigated as follows: (1) The CBF value of each ROI was extracted from each subject’s CBF map. (2) CBFC between each seed ROI and all other voxels of the brain across individuals was calculated by multiple regression models using sex and age as covariates, and brain regions whose CBF values were significantly positively or negatively correlated with the CBF value of each seed ROI were identified. (3) The CBFC graphs of the same ROI of two groups were combined into a spatial mask. The generated mask contained all the voxels whose CBF was significantly correlated with the CBF of the seed ROI. (4) To identify the voxels with significantly different CBF correlation coefficients within the generated mask, the two-sample t test was then run on the CBF correlogram within the mask of the same ROI in the two groups after controlling for age, sex, and years of education. All statistical maps were corrected for multiple comparisons by AlphaSim correction with a voxel-level *p* < 0.001, AlphaSim value < 0.05.

## Results

### Demographic characteristics and ophthalmological and neuropsychological findings

Differences in demographic characteristics and ophthalmological and neuropsychological measurements between POAG patients and NCs were assessed by SPSS 23 (V.23.0. Chicago, Illinois, SA). The independent-samples t-test was used to analyze the group differences in quantitative data (age, BDI score, STAI-T/S score, MoCA score and SDMT score), and the chi-square test was applied to analyze the group differences in sex. Data were considered significant at *p* < 0.05. There were no significant intergroup differences in age, sex, MoCA, or SDMT. Patients showed lower MD and larger PSD and CDR than NCs, and the BDI score and STAI score (state and trait components) of POAG patients were significantly higher than those of NCs (Table 1).

### CBF changes and its correlation with ophthalmological and neuropsychological assessments in patients with POAG

**Fig. 1:**
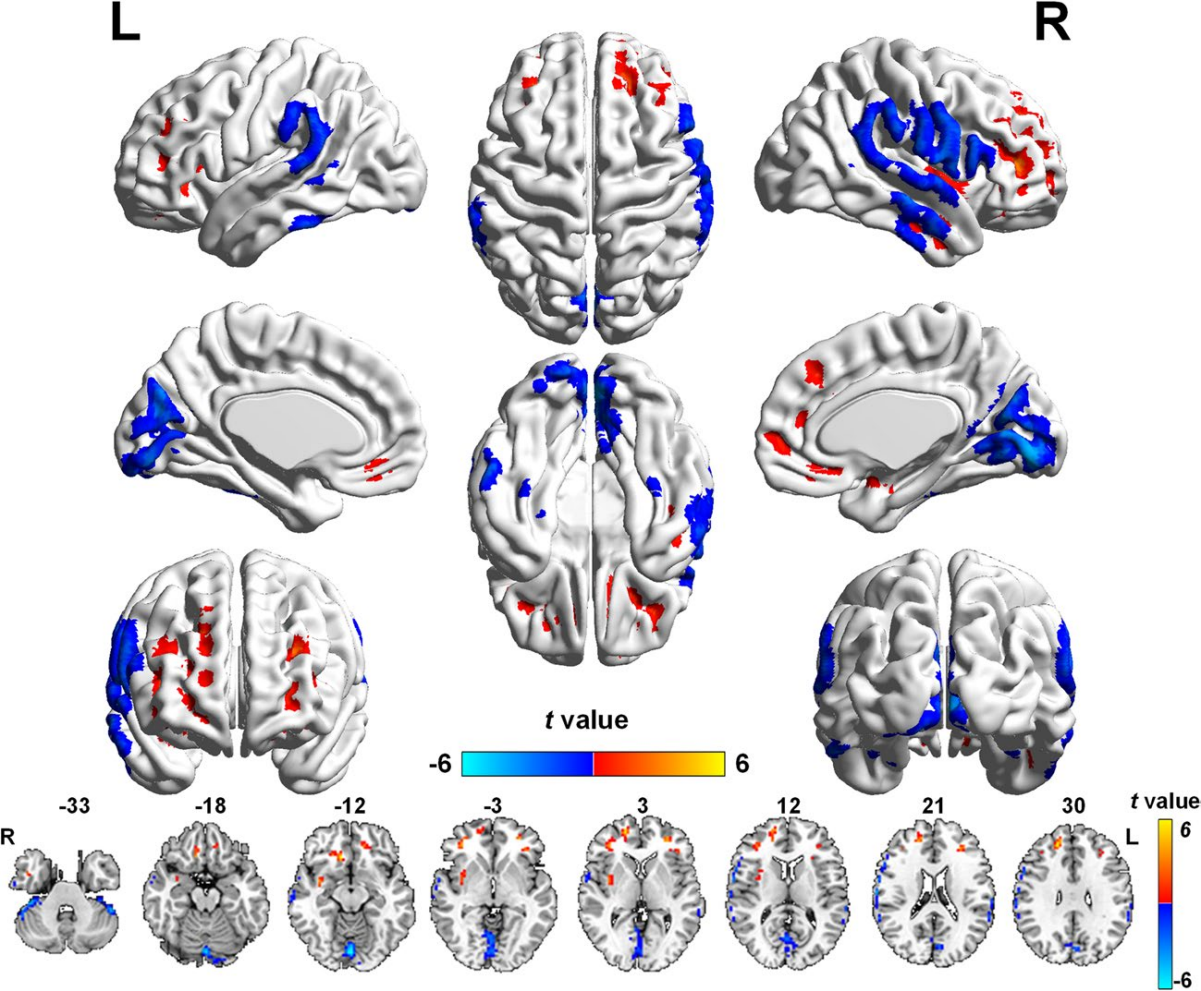
Brain regions with altered CBF values in POAG patients compared with NCs. The cold color represents the decreased CBF in POAG patients are located in the bilateral LG and Cal; a region centered in the right PostCG and extending to the right IFG, SMG and IPL; a region extending from the IPL to the left SMG; the left cerebellar crus I; and the right cerebellar lobule VI. The warm color denotes the increased CBF values in POAG patients are set in the right medial FG; a region extending from the left MFG to the left medial prefrontal cortex; the bilateral MFG; the right SFG; and the right insula. (GRF-corrected voxel *p* value < 0.001 and cluster *p* value < 0.05). Abbreviations: CBF, cerebral blood flow; NCs, normal controls; POAG, primary open-angle glaucoma; LG, lingual gyri; Cal, calcarine gyri; PostCG, postcentral gyrus; IFG, inferior frontal gyrus; SMG, supramarginal gyrus; IPL, inferior parietal lobule; Medial FG, Medial frontal gyrus; MFG, middle frontal gyrus; SFG, superior frontal gyrus; GRF, Gaussian Random Field Theory

Compared with the NCs, the POAG patients showed decreased CBF in the bilateral lingual gyrus (LG) and calcarine gyrus(Cal) (t = −5.654), right postcentral gyrus (PostCG) (t = −4.643) and inferior parietal lobule(IPL) (t = −3.951), and left cerebellar crus I (t = −4.673)and right cerebellar lobule VI (t = −4.678); and increased CBF in the right medial prefrontal gyrus and medial frontal gyrus(Medial FG) (t = 4.857), left middle frontal gyrus (MFG) (t = 4.525), the right (t = 4.563) and left MFG(t = 4.394), the right superior frontal gyrus(SFG) (t = 5.137), and the right insula(t = 4.441) (Fig. 1 and Table 2). In addition, we repeated the analysis with gray matter volume as a covariate and obtained similar results with the main findings, shown in Supplementary Figure S1.

**Table 2:** Brain regions with significant differences of CBF values between POAG patients and NCs

Brain regions	Voxels	Peak MNI coordinates(mm)			Peak intensity
		x	y	z	
POAG < NC					
B_LG and Cal	395	0	−84	−12	−5.654
R_PostCG	277	66	−15	21	−4.643
L_IPL	57	−63	−45	21	−3.951
L_CereCrus1	61	−51	−45	−33	−4.673
R_Cere_6	58	39	−33	−33	−4.678
POAG > NC					
R_Ins	99	36	−3	−24	4.441
R_Medial FG	36	12	27	−12	4.857
L_MFG and Medial FG	40	−30	48	0	4.525
L_MFG	62	−30	36	24	4.394
R_MFG	90	36	39	0	4.563
R_SFG	146	18	57	9	5.137

GRF correction voxel *p* value < 0.001, cluster *p* < 0.05

Abbreviations: *CBF* cerebral blood flow, *POAG* primary open angle glaucoma, *NCs* normal controls, *GRF* Gaussian random field theory, *R* right, *L* left, *B* bilateral, *LG* lingual gyri, *Cal* calcarine gyri, *PostCG* postcentral gyrus, *IPL* inferior parietal lobule, *Cere* cerebellar, *Ins* insula, *Medial FG* medial frontal gyrus, *MFG* middle frontal gyrus, *SFG* superior frontal gyrus

After correction for multiple comparisons (Bonferroni correction, *p* < 0.05/11 = 0.005), the reduced CBF in the bilateral LG and Cal positively correlated with MD of VF defect (*r* = 0.498, *p* = 0.001) in POAG patients. The decreased CBF in the left cerebellar crus I had a trend of negative correlation with MD of VF defect (*r* = 0.376, *p* = 0.015). While the increased CBF in the right Medial FG was negatively associated with the MD of VF defect (*r* = −0.438, *p* = 0.004) and positively associated with the CDR (*r* = 0.469, *p* = 0.002). For the neuropsychological assessments, the significance of the correlations did not remain after Bonferroni correction (Table 3).

**Table 3:** Correlations between CBF values and ophthalmological examination, and neuropsychological assessments in POAG

Regions	MD	CDR	STAI-S	STAI-T	BDI	MoCA	SDMT
t-value(*P*-value)							
B_LG and Cal	0.498**(0.001)	−0.125(0.442)	−0.314*(0.043)	− 0.193(0.221)	−0.287(0.069)	0.144(0.363)	0.055 (0.763)
R_PostCG	0.028(0.863)	−0.115(0.478)	−0.064(0.688)	− 0.074(0.643)	0.036(0.823)	0.225(0.153)	−0.045 (0.805)
L_IPL	0.265(0.094)	−0.275(0.086)	0.012(0.939)	− 0.015(0.923)	−0.012(0.942)	0.196(0.214)	0.140 (0.436)
R_Cere_6	0.044(0.782)	0.185(0.252)	−0.147(0.354)	− 0.206(0.191)	0.018(0.911)	−0.214(0.174)	−0.284 (0.109)
L_CereCrus1	0.376*(0.015)	−0.168(0.299)	−0.232(0.14)	− 0.206(0.191)	0.049(0.761)	−0.008(0.958)	−0.222 (0.215)
R_Ins	−0.306(0.052)	0.1(0.538)	0.25(0.111)	0.218(0.166)	0.264(0.095)	−0.237(0.131)	−0.224 (0.210)
R_Medial FG	−0.438**(0.004)	0.469**(0.002)	0.108(0.497)	0.161(0.309)	−0.025(0.876)	−0.233(0.137)	0.018 (0.922)
L_MFG and Medial FG	−0.164(0.306)	0.107(0.51)	0.062(0.696)	0.217(0.167)	0.132(0.41)	0.059(0.711)	0.127 (0.480)
L_MFG	−0.186(0.243)	0.054(0.74)	0.324*(0.036)	0.412*(0.007)	0.331*(0.034)	−0.02(0.898)	−0.055 (0.759)
R_MFG	−0.22(0.166)	0.253(0.116)	0.089(0.575)	0.116(0.465)	0.045(0.78)	−0.04(0.78)	0.210 (0.242)
R_SFG	−0.22(0.168)	0.297(0.063)	−0.026(0.871)	0.015(0.926)	−0.179(0.262)	0.062(0.698)	0.254 (0.153)

Abbreviations: *CBF* cerebral blood flow, *POAG* primary open angle glaucoma, *MD* mean deviation of visual field defects, *CDR* cup-to-disc ratio, *STAI-S and STAI-T* State-trait anxiety inventory, *BDI* beck depression inventory. *MoCA* Montreal cognitive assessment (MoCA), *SDMT* symbol digit modalities test, *R* right, *L* left, *LG* lingual gyri, *Cal* calcarine gyri, *PostCG* postcentral gyrus, *IPL* inferior parietal lobule, *Cere* cerebellar, *Ins* insula, *Medial FG* medial frontal gyrus, *MFG* middle frontal gyrus, *SFG* superior frontal gyrus

^*^p < 0.05, **p < 0.005

### CBFC patterns and group differences in CBFC

**Fig. 2:**
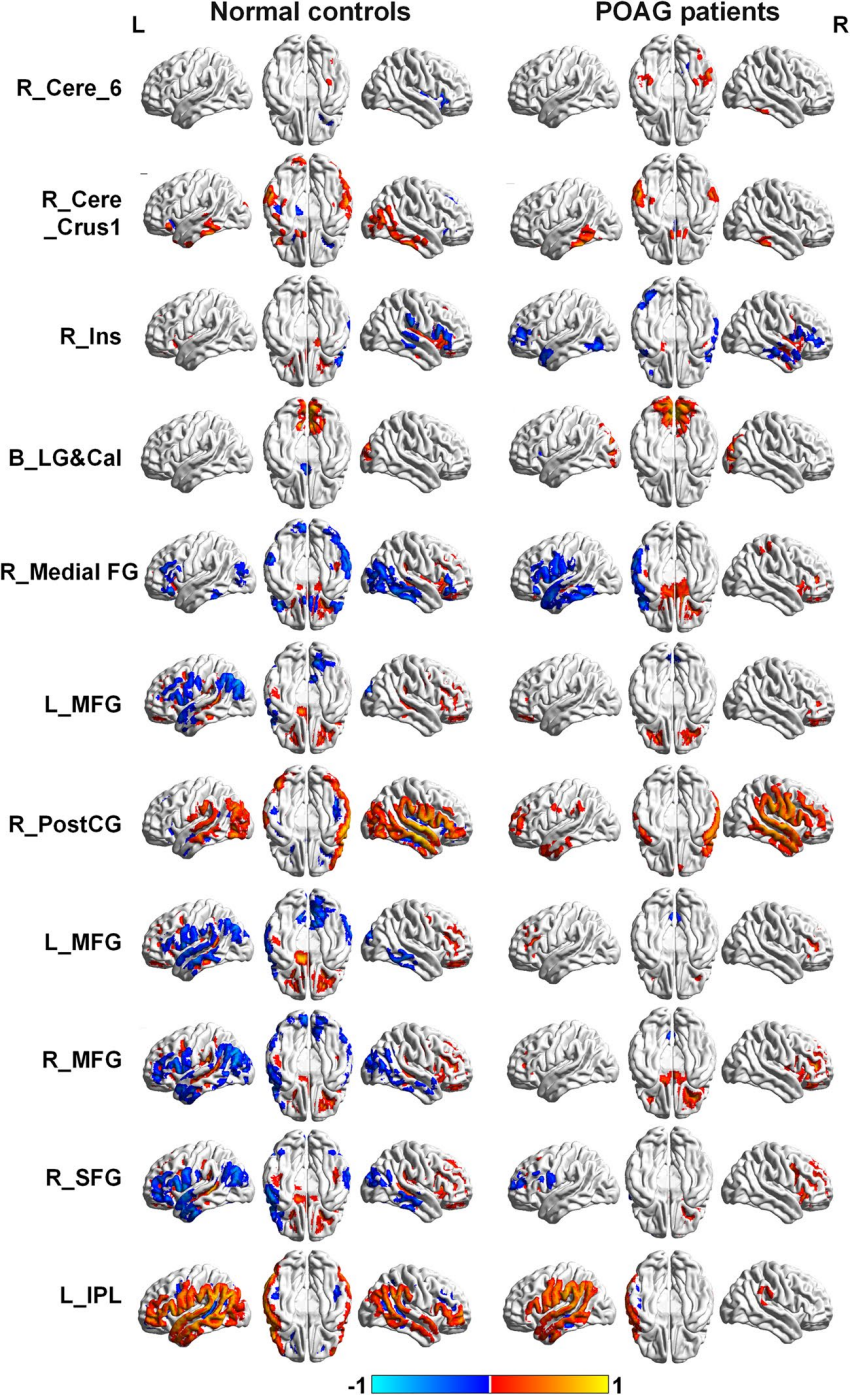
The CBF connectivity maps of eleven ROIs from NCs and POAG patients. The The warm color indicated positive connections, and cold color indicated negative connections (AlphaSim correction with a voxel-level *p* < 0.001, AlphaSim value < 0.05). Abbreviations: POAG, primary open angle glaucoma; NCs, normal controls; GRF, Gaussian Random Field Theory. R, right; L, left; B, bilateral; LG, lingual gyri; Cal, calcarine gyri; PostCG, postcentral gyrus; IPL, inferior parietal lobule; Cere, cerebellar; Ins, insula; Medial FG, Medial frontal gyrus; MFG, middle frontal gyrus; SFG, superior frontal gyrus

**Fig. 3:**
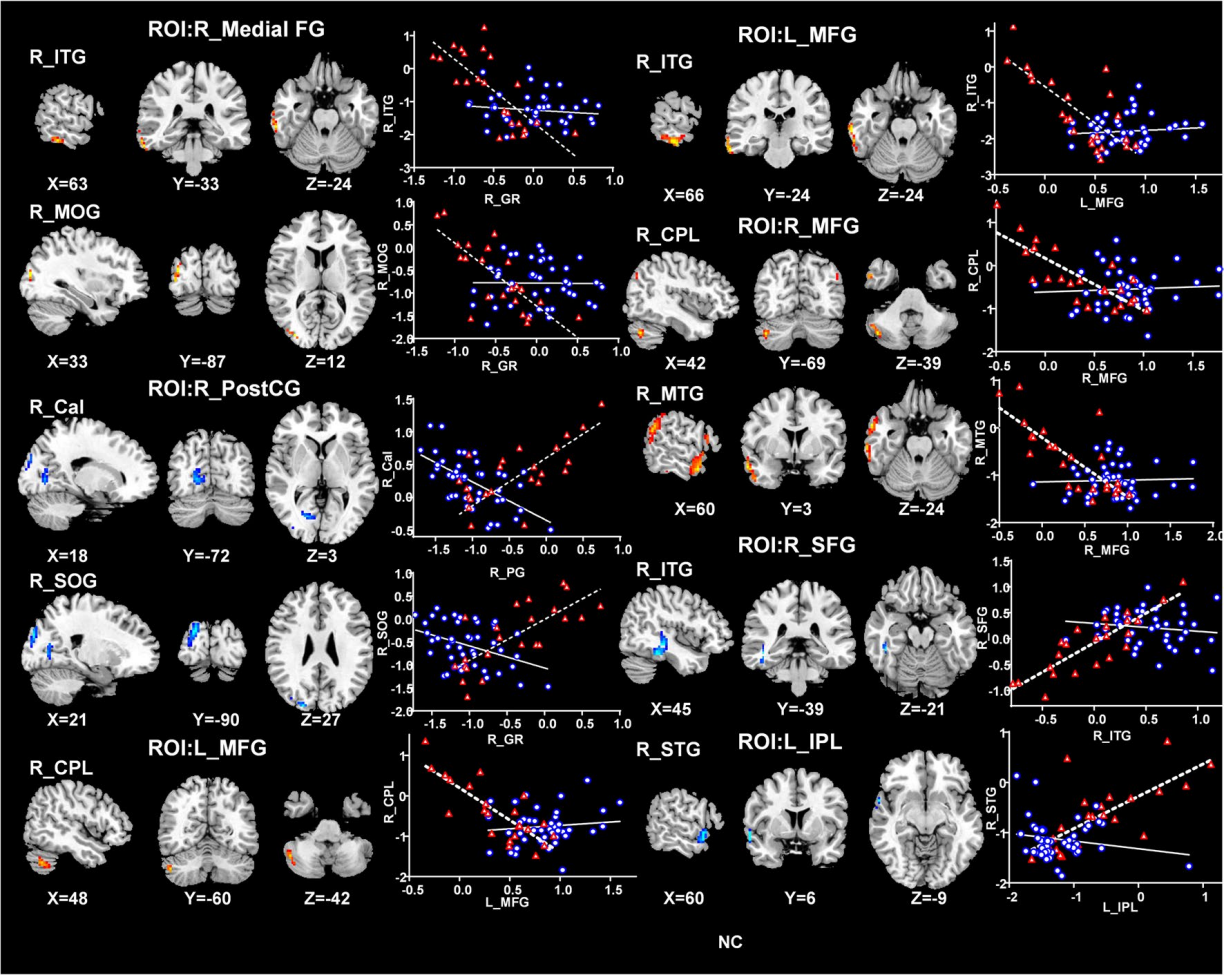
Brain regions with significant CBF connectivity differences between NCs and POAG patients. Compared with the NCs (dashed line), the POAG patients (solid line) showed decreased negative CBF connectivity between right Medial FG and right ITG as well as right MOG. Besides, contrary to the NCs, the POAG patients showed negative CBF connectivity between right PostCG and right Cal as well as right SOG, between right SFG and right ITG, and left IPL and right STG, and showed positive CBF connectivity between left MFG and right CPL as well as right ITG and between right MFG and right CPL as well as right MTG. Abbreviations: CBF, cerebral blood flow; POAG, primary open angle glaucoma; NCs, normal controls; R, right; L, left; Medial FG, Medial frontal gyrus; ITG, inferior temporal gyrus; MOG, middle occipital gyrus; PostCG, postcentral gyrus; Cal, Calcarine; SOG, superior occipital gyrus; SFG, superior frontal gyrus; STG, superior temporal gyrus; IPL, inferior parietal lobule; MFG, middle frontal gyrus; CPL, cerebellar posterior lobe; MTG, middle temporal gyrus

Eleven brain regions with significant CBF differences between the POAG patients and NCs were obtained as seed ROIs. Figure 2 displayed the CBFC maps (GRF correction combining a voxel-level *p* value < 0.001 and cluster-level *p* < 0.05) of each ROI in the two groups, and the two groups showed different CBFC patterns. Compared with the NCs, POAG patients showed decreased negative CBFC between the seed ROI of right medial prefrontal cortex and right inferior temporal gyrus (ITG) (*r*_*NCs*_ = −0.734, *p*_*NCs*_ < 0.001 & *r*_*POAG*_ = −0.122, *p*_*POAG*_ = 0.431) as well as right middle occipital gyrus (MOG) (*r*_*NCs*_ = −0.744, *p*_*NCs*_ < 0.001 & *r*_*POAG*_ = −0.016, *p*_*POAG*_ = 0.917). In contrast to the NCs, the POAG patients showed negative CBFC between the seed ROI of the right PostCG and right Cal (*r*_*NCs*_ = 0.681, *p*_*NCs*_ < 0.001 & *r*_*POAG*_ = −0.613, *p*_*POAG*_ < 0.001) as well as right superior occipital gyrus (SOG) (*r*_*NCs*_ = 0.737, *p*_*NCs*_ < 0.001 & *r*_*POAG*_ = −0.400, *p*_*POAG*_ = 0.007), between the seed ROI of right SFG and right ITG (*r*_*NCs*_ = 0.797, *p*_*NCs*_ < 0.001 & *r*_*POAG*_ = −0.132, *p*_*POAG*_ = 0.393), and between the seed ROI of left IPL and right superior temporal gyrus (STG) (*r*_*NCs*_ = 0.677, *p*_*NCs*_ = 0.001 & *r*_*POAG*_ = −0.186, *p*_*POAG*_ = 0.226). The patients showed positive CBFC between the seed ROI of left MFG and right cerebellar posterior lobe (CPL) (*r*_*NCs*_ = −0.784, *p*_*NCs*_ < 0.001 & *r*_*POAG*_ = 0.119, *p*_*POAG*_ = 0.441)as well as right ITG (*r*_*NCs*_ = −0.749, *p*_*NCs*_ < 0.001 & *r*_*POAG*_ = 0.095, *p*_*POAG*_ = 0.538)and between the seed ROI of right MFG and right CPL (*r*_*NCs*_ = −0.770, *p*_*NCs*_ < 0.001 & *r*_*POAG*_ = 0.063, *p*_*POAG*_ = 0.683) as well as right middle temporal gyrus (MTG) (*r*_*NCs*_ = −0.743, *p*_*NCs*_ < 0.001 & *r*_*POAG*_ = 0.037, *p*_*POAG*_ = 0.813) (Fig. 3).

## Discussion

The results of this study demonstrate that brain regions with redistributed cerebral blood in patients with POAG were not confined to the visual system. In patients with POAG, the bilateral LG and Cal, righ PostCG, left IPL, and left cerebellar crus I and right cerebellar lobule VI showed decreased CBF. These brain areas are the key hubs of DMN, DAN, somatosensory network and cerebellar networks with higher neuronal activity need higher energy (Fu et al., 2019; Stoodley & Schmahmann, 2018), they may be highly susceptible to conditions that endanger their energy supply and become primary targets of neurodegenerative diseases (Tomasi et al., 2013).While brain regions related to high-level cognition such as attention, emotion, thought inhibition, motor control, etc., including the right medial prefrontal gyrus, MFG, SFG and insula, bilateral MFG, as well as left MFG extending to the left medial prefrontal gyrus, showed increased CBF in patients with POAG. Additionally, we found significant correlations between the CBF changes and glaucomatous visual functional and structural damage. The abnormal CBF in visual cortices and left MFG in POAG patients tended to correlate with anxiety and depression scores. Another important result of this study is that the CBFC of the right Medial FG, PostCG and SFG, left IPL and bilateral MFG was impaired in POAG. Overall, these findings corroborate the hypothesis of the spreading neurodegeneration in POAG with the brain-wide regional and interregional CBF properties abnormalities extending beyond visual pathway, including the somatosensory, emotional and cognitive networks.

Hypoperfusion in the bilateral LG and Cal contribute to the deficits in visual perception and the integration of visual information in POAG patients. The Cal and LG situated in the primary visual cortex (V1) and visual association cortices (V2), respectively. V1 receives afferent fibers from the lateral geniculate body while transmitting information to the visual association cortices (Di Cio et al., 2020). V2 is the only way for the V1 to output information to the ventral and dorsal visual streams (Di Cio et al., 2020). The neural input to the visual cortices is reduced because of the glaucomatous damage to the retinal ganglion cells and the VF defects. Lower visually evoked metabolic activation, and decreased functional activity of visual cortices in POAG have been observed in neuroimaging studies (Kasi et al., 2019). According to the neurovascular unit theory, it can be speculated the decreased CBF in the calcarine and lingual gyri may be due to deprivation of visual input and transsynaptic degeneration. In line with the previous papers of our team, POAG patients showed reduced CBF and reduced CBF/FCS ratio in visual cortices (Wang et al., 2018; Wang et al., 2021a). In addition, those studies as well as the present one found the more severe the VF defect, the lower the CBF in visual cortex, which confirmed the clinical relevance of the CBF changes in visual cortices of POAG patients.

Changes in PostCG, IPL, and posterior lobe of cerebellum in patients with glaucoma have been demonstrated in morphological and functional studies (Chen et al., 2013; Dai et al., 2013; Hanafiah et al., 2022; Jiang et al., 2018, 2019). The PostCG, acting as the hub for somatosensory network, has a wide range of physiological connections with the visual cortex and primary motor cortex (Fu et al., 2019). It may be involved in encoding for the visual motion related to changing direction of self-motion in the environment, and its sensitivity can be modulated by visual input (Di Marco et al., 2021). The cerebellar crus I and right cerebellar lobule VI are engaged in visuospatial, visual motor and cognitive function (Ashida et al., 2019; Stoodley & Schmahmann, 2018; Sugihara, 2018). The IPL is the key component of in the DMN, which governs shifts of spatial attention and target detection, dealing with visuospatial recognition and coordination and guiding movement (Crottaz-Herbette et al., 2014; Nucci et al., 2020). It has been shown that POAG patients encountered difficulties in visuomotor coordination, orientation and dividing attention, increasing the risk of falling and unsafe driving, point toward a link between POAG and changes in PostCG, IPL, and posterior lobe of cerebellum (Di Cio et al., 2020; Montana & Bhorade, 2018).

It is important to note that glaucoma can cause psychological disturbances in patients, including anxiety and depression, even at an early stage (Wu et al., 2022). The PostCG and IPL are the critical regions of primary somatosensory cortex (S1) and the secondary somatosensory cortex (S2), respectively (Kropf et al., 2019). They are involved in each stage of emotional processing and play a central role in the regulation of emotions (Kropf et al., 2019). Situations that resulted in negative emotions caused decreased activity in somatosensory cortex (Kropf et al., 2019). In agreement with previous report of the increased GMV of the right insula in glaucoma patients (Jiang et al., 2018), we found increased CBF in right insula. The insula connecting with the cingulate, frontal, parietal and sensorimotor areas subserves a wide variety of functions in humans, including the emotional regulation, and it can be activated by negative emotional stimuli (Uddin et al., 2017). The increased CBF of medial FG indicated increased excitability, and the results indicated that the more severe the glaucoma is, the higher the perfusion in the medial FG. As a result, the excitation/inhibition balance in the medial FG was impaired, which is considered one of the pathogenic mechanisms for anxiety disorders (Xu et al., 2019). These findings are in accordance with the existing evidence that POAG patients are more likely to develop depression and anxiety disorders than NCs (Zhang et al., 2021).

Emotion and attention interact; patients with depression and anxiety usually show attentional bias toward unfavorable or negative stimuli, and individuals' attentional bias toward negative stimuli is an important factor inducing mood fluctuations, which will aggravate patients' emotional disorders (Fajkowska et al., 2018). The MFG implicated in attentional reorientation showed higher CBF in POAG patients (Briggs et al., 2021), and we speculated the increased CBF in MFG might be related to its enhanced attentional bias towards to negative stimuli. Compared with NCs, glaucoma patients have had increased CBF in the SFG, which is consistent with the findings of higher activity of the SFG in glaucoma patients, and the amplitude of low-frequency fluctuation (ALFF) of the SFG was positively correlated with disease severity (Li et al., 2014; Wang et al., 2021b). We inferred that there may be two possible causes of the hyperperfusion in SFG. First, as a key node of the frontoparietal control network, SFG plays a role in thought suppression, which is essential for the inhibition of negative emotions (Lu et al., 2022). Here, the increased CBF in SFG was intended to reduce patients' anxiety and depressive symptoms. Second, POAG patients perform more saccades than NCs to compensate for VF loss, and also a result of the neurodegeneration (Najjar et al., 2017). SFG is the important region implicated in eye movement, and simulation of the SFG can induce saccadic eye movements (Briggs et al., 2020). The hyperperfusion in SFG may contribute to their aberrant eye movements. Unfortunately, our results did not show significant correlations between the CBF changes and the neuropsychological assessments after Bonferroni correction, and we did not evaluate the attentional bias in these subjects. In the future, a combination of emotional functional MRI and the Attention to Positive and Negative Information scale will be used to investigate this phenomenon.

The changes in regional CBF values in different brain regions are not independent. We further explored the CBFC alterations in these brain regions in POAG. The CBFC indicates different physiological meanings from BOLD connectivity, which reflects neural activity synchronized over time between brain regions, CBFC reflects coordinated metabolism or perfusion between brain regions. In contrast to BOLD connectivity, which is affected by multiple physiological factors, the CBFC is modulated only by regional CBF and has a more specific physiological significance (Melie-Garcia et al., 2013). Compared with NCs, the POAG patients showed disordered connection modes, mainly characterized by some negative connections decreased or transformed into mild positive connections, and positive connections converted to negative connections. For the medial FG, the POAG patients showed decreased CBFC in ipsilateral ITG and MOG, and for the bilateral MFG, the POAG patients showed positive CBFC in right CPL, ITG, and MTG, when compared to the NCs. These brain regions perform visual, emotional, and cognitive functions, and the activated brain connections may convey top-down regulation from the higher cortical regions in response to visual impairment, emotional abnormalities, and cognitive impairment. Conversely, for the PostCG, SFG, and IPL, the positive connectivities converted into negative connectivities in right Cal, SOG, ITG, and STG in patients with POAG compared with NCs. These regions with lower CBFC are involved in visual network, working memory, and DAN, in line with previous BOLD connectivity analyses that identified reduced functional connectivity in these networks in POAG patients (Frezzotti et al., 2014). These deactivated interregional connectivities indicated the functional deficits in integration of visual information, visuospatial location, and visual movements.

The current study has several limitations: its sample size, behavioral data, and cross-sectional design, as well as methodological limitations and challenges. First, the current sample size of this study is not sufficient to investigate the pattern of cerebral perfusion changes in different disease stage of POAG. The study is ongoing, and we will expand the sample size to conduct subgroup analysis to explore the trend of CBF changes as disease progresses. Second, the abnormal cerebral perfusion in POAG patients involved not only the visual association cortex but also brain regions in emotional and attentional networks. However, this study was not able to directly confirm the relationship between altered cerebral perfusion and abnormal behavior. Future research will combine emotional functional MRI with the Attention to Positive and Negative Information scale to investigate the relationship. Third, this is a cross-sectional study, and we did not regularly follow up the enrolled patients with functional MRI, which could not clarify the causal relationship between the behavioral abnormalities and changes in CBF. To determine the causal relationship between CBF changes and glaucoma with sufficient statistical power, longitudinal measurements of a large sample are necessary. Finally, when investigating the features of CBFC alterations in brain regions with significant group differences, we ignore changes in CBFC between regions with normal CBF. There is a need for a data-driven method to analyze CBFC throughout the entire brain.

In conclusion, disturbed CBF in patients with POAG extensively involved brain regions beyond the visual pathway including the somatosensory network, frontoparietal control network, working memory network, and DAN, and have the potential clinical and behavioral relevance. The extended findings of the present study demonstrated that the analysis conducted by the CBFC revealed interdependencies among regional CBF values in various brain regions, indicating co-variation properties within functional networks. This study highlights the importance of combining regional CBF and interregional CBFC in exploring the underlying mechanism of neurodegeneration spreading in patients with POAG.

### Supplementary Information

Below is the link to the electronic supplementary material.

Supplementary file1 (DOCX 800 KB)

## Data Availability

The data and materials of this study are available from the corresponding author on reasonable request.
