# Supplementary material for: Arterial spin labeling reveals disordered cerebral perfusion and cerebral blood flow-based functional connectivity in primary open-angle glaucoma
Source: Brain Imaging Behav. 2023 Nov 25;18(1):231–42. doi: 10.1007/s11682-023-00813-2 (PMC10844339; doi:10.1007/s11682-023-00813-2)
Supplement: Supplementary file 1 — Supplementary file1 (DOCX 800 KB) [file 11682_2023_813_MOESM1_ESM.docx]

**Supplementary figure legend**
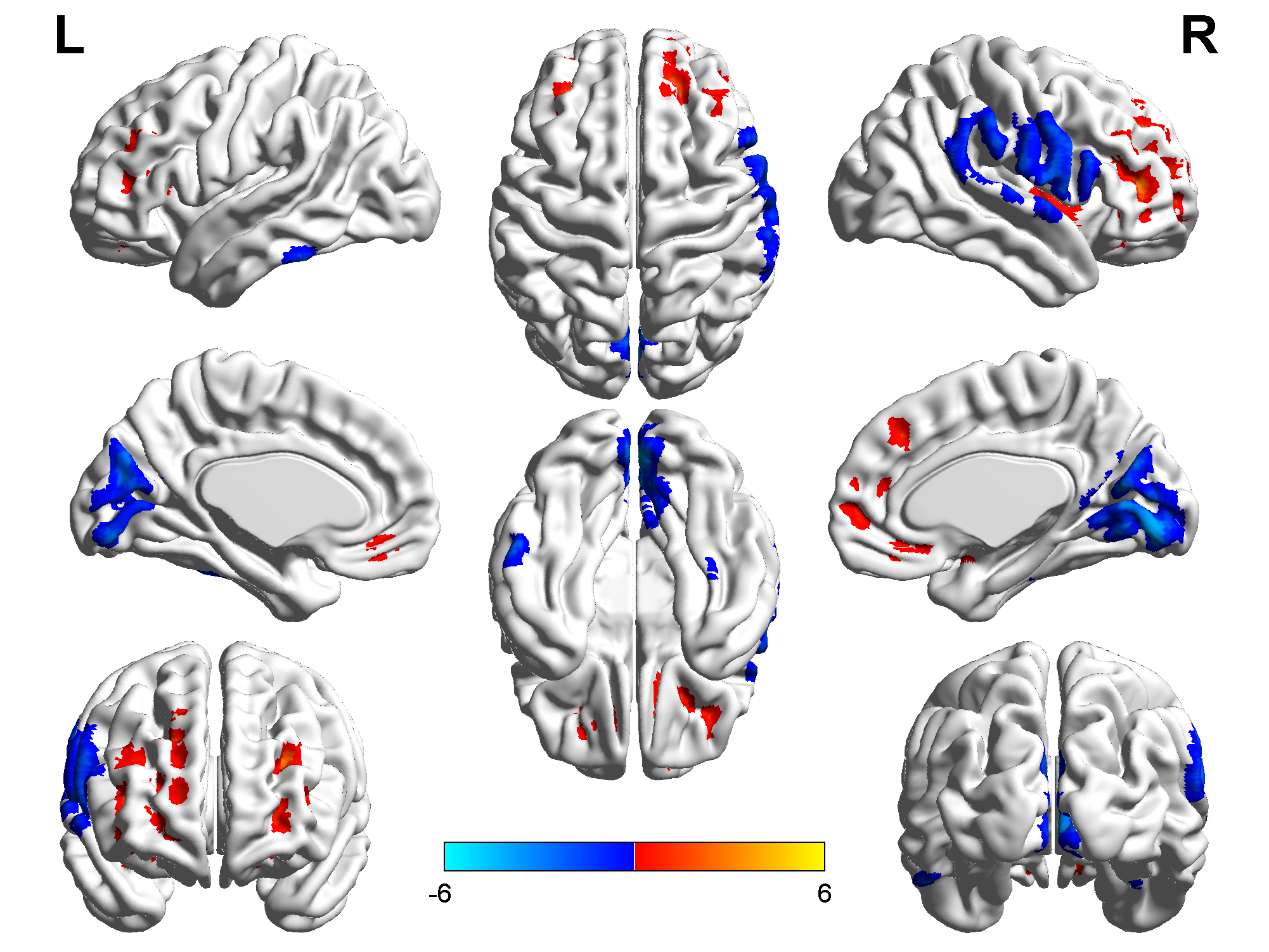


**Supplementary Figure S1.** Brain regions with significant differences in CBF values in POAG patients compared with NCs with GMV as a covariate. The warm and cold color represents significantly increased and decreased CBF value, respectively. The POAG patients showed significantly decreased CBF in the bilateral LG, bilateral Cal, right PostCG, left IPL, and bilateral cerebellum, as well as increased CBF values in the right medial FG, a region extending from the left MFG to the left medial prefrontal cortex, the bilateral MFG, and the right SFG, whose peak MNI coordinates were the same as those of the regions without GMV correction. These findings suggest that altered CBF values in these regions in POAG patients are independent of GMV changes. However, the peak MNI coordinate (x/y/z =36/-3/-24) of the original cluster with increased CBF in the right insula changed to x/y/z =36/0/-12, which indicated that the altered CBF in the right insula was related to GMV. (GRF-corrected voxel *p* value<0.001 and cluster *p* value <0.05).

Abbreviations: CBF, cerebral blood flow; NCs, normal controls; POAG, primary open-angle glaucoma; GMV, gray matter volume; LG, lingual gyri; Cal, calcarine gyri; PostCG, postcentral gyrus; IPL, inferior parietal lobule; Medial FG, Medial frontal gyrus; MFG, middle frontal gyrus; SFG, superior frontal gyrus; GRF, Gaussian Random Field Theory.
